# Fine-Scale Reconstruction of the Evolution of FII-33 Multidrug Resistance Plasmids Enables High-Resolution Genomic Surveillance

**DOI:** 10.1128/msystems.00831-21

**Published:** 2022-01-18

**Authors:** Ya Hu, Robert A. Moran, Grace A. Blackwell, Alan McNally, Zhiyong Zong

**Affiliations:** a Center of Infectious Diseases, West China Hospital, Sichuan University, Chengdu, China; b Institute of Microbiology and Infection, College of Medical and Dental Sciences, University of Birmingham, Birmingham, United Kingdom; c EMBL-EBI, Wellcome Genome Campus, Hinxton, United Kingdom; d Wellcome Sanger Institute, Wellcome Genome Campus, Hinxton, United Kingdom; e Department of Infection Control, West China Hospital, Sichuan University, Chengdu, China; f Center for Pathogen Research, West China Hospital, Sichuan University, Chengdu, China; g Division of Infectious Diseases, State Key Laboratory of Biotherapy, Chengdu, China; Dalhousie University

**Keywords:** *Enterobacteriaceae*, *Klebsiella*, antibiotic resistance, carbapenem resistance, plasmid typing, plasmids, surveillance

## Abstract

We examined 185 complete, publicly available FII-33 plasmid sequences, characterizing their backbone and various insertions. The variable characteristic insertions facilitated evolutionary reconstruction for this plasmid group, beginning with the acquisition of a primary resistance region (PRR) over 10 years ago. FII-33 plasmids have evolved by acquiring additional resistance genes in the PRR via translocatable elements and by forming cointegrates with plasmids of other types. In all cases, IS*26* is suspected to have mediated cointegration. Plasmid cointegration has contributed to the accumulation of resistance genes and may have increased the transmissibility, stability, and host range of the original FII-33 lineage. A particularly important sublineage was formed by a replicative IS*26* cointegration event that fused an FII-33 plasmid with a *bla*_KPC-2_-containing R-type plasmid, interrupting the FII-33 *traI* gene encoding the conjugative relaxase. The FII-33:R cointegrate arose in the Klebsiella pneumoniae ST11 clone and remains largely confined there due to the abolition of transfer ability by the FII-33:R cointegration event. However, in some cases FII-33:R cointegrates have fused with additional plasmids and acquired complete transfer regions or *oriT* sequences that might restore their ability to transfer horizontally. Cointegration events across FII-33 plasmid sublineages have involved plasmids of at least 15 different types. This suggests that plasmid cointegration occurs readily and is more common than previously appreciated, raising questions about the effects of cointegrate formation on plasmid host range, stability, and capacity for horizontal transfer. Resources are provided for detecting and characterizing FII-33 plasmid sublineages from complete or draft genome sequences.

**IMPORTANCE** Effective genomic surveillance of antibiotic-resistant bacterial pathogens must consider plasmids, which are frequently implicated in the accumulation and transfer of resistance genes between bacterial strains or species. However, the evolution of plasmids is complex, and simple typing or comparison tools cannot accurately determine whether plasmids belong to the same sublineages. This precludes precise tracking of plasmid movement in bacterial populations. We have examined the FII-33 group, which has been associated with multidrug resistance and particularly carbapenem resistance in clinically significant members of the *Enterobacterales* in China. Our analysis has provided insight into the evolution of this important plasmid group, allowing us to develop resources for rapidly typing them to the sublineage level in complete or draft genome sequences. Our approach will improve detection and characterization of FII-33 plasmids and facilitate surveillance within and outside China. The approach can serve as a model for similar studies of other plasmid types.

## INTRODUCTION

The important role played by plasmids in the spread of antibiotic resistance genes into and between bacteria responsible for human infections is well appreciated. However, the evolution and dissemination of individual plasmid lineages is poorly understood, as basic typing methods do not provide insight into the complex mechanisms by which plasmids acquire, lose, or substitute genetic material as they move between geographic locations and bacterial populations. Tracking individual plasmid lineages will be crucial for surveillance of antibiotic resistance determinants and for furthering our understanding their spread.

F-type plasmids were the first to be described, beginning with the discovery of the conjugative plasmid F by Esther Lederberg in the 1950s (1). Although plasmid F did not confer resistance to antibiotics, the F-like plasmids NR1 (also called R100) from Japan and R1 from the United Kingdom, isolated in the 1950s and 1960s, respectively, contained multiple antibiotic resistance genes in a complex region that is called Tn*2670* in NR1 (2). F-type plasmids have since been found throughout the *Enterobacterales* (3) and shown by many studies to be the most common plasmid type in Escherichia coli from human clinical (4), human commensal (5), agricultural (6, 7), and environmental sources. Like antibiotic resistance genes, F-type plasmids can carry virulence, iron acquisition, and colicin genes (8), potentially contributing to their hosts’ ability to compete in gastrointestinal environments or facilitating growth at extraintestinal human body sites.

Many F-type plasmid replicons have been identified, including variants of FIA, FIB, and FII types that occur alone or in combination. The FII-33 replicon was first associated with the antibiotic resistance genes *bla*_TEM_, *bla*_CTX-M-65_, *fosA3*, and *rmtB* in an Escherichia coli isolated in China in 2008 (9). Sporadic reports since have described FII-33 plasmids carrying antibiotic resistance genes in *Enterobacterales* isolated from clinical (10) and agricultural (11) settings as well as from pets (12). While almost all descriptions of FII-33 plasmids have come from China, they have also been reported in clinical Klebsiella pneumoniae isolates in Bolivia, indicating that there has been some international dissemination. More recently, FII-33 plasmids have been reported to have formed cointegrates with other types of plasmids (13–15). In Sichuan, China, FII-33 plasmids have been responsible for β-lactam, aminoglycoside, chloramphenicol, fosfomycin, tetracycline, and quinolone resistance in K. pneumoniae involved in extensive hospital outbreaks (16).

Here, we have performed a comprehensive analysis of the FII-33 plasmid group by examining 20 complete plasmid sequences derived from clinical K. pneumoniae and E. coli isolates collected in Sichuan province between 2014 and 2018 and a further 165 complete, publicly available sequences. Through comparative analyses, we have traced the evolutionary history of the group and defined sublineages by events that introduced translocatable elements to the FII-33 backbone. Within sublineages, we have traced the more recent events that have resulted in the accumulation of resistance genes, formation of cointegrates, and loss or diversification of FII-33 backbone sequences. This has provided general insights into the mechanisms by which plasmids evolve while circulating in bacterial populations. Materials provided here will enable rapid typing of FII-33 sublineages and facilitate ongoing surveillance of this important plasmid group.

## RESULTS AND DISCUSSION

### FII-33 plasmids are endemic to China.

**FIG 1:**
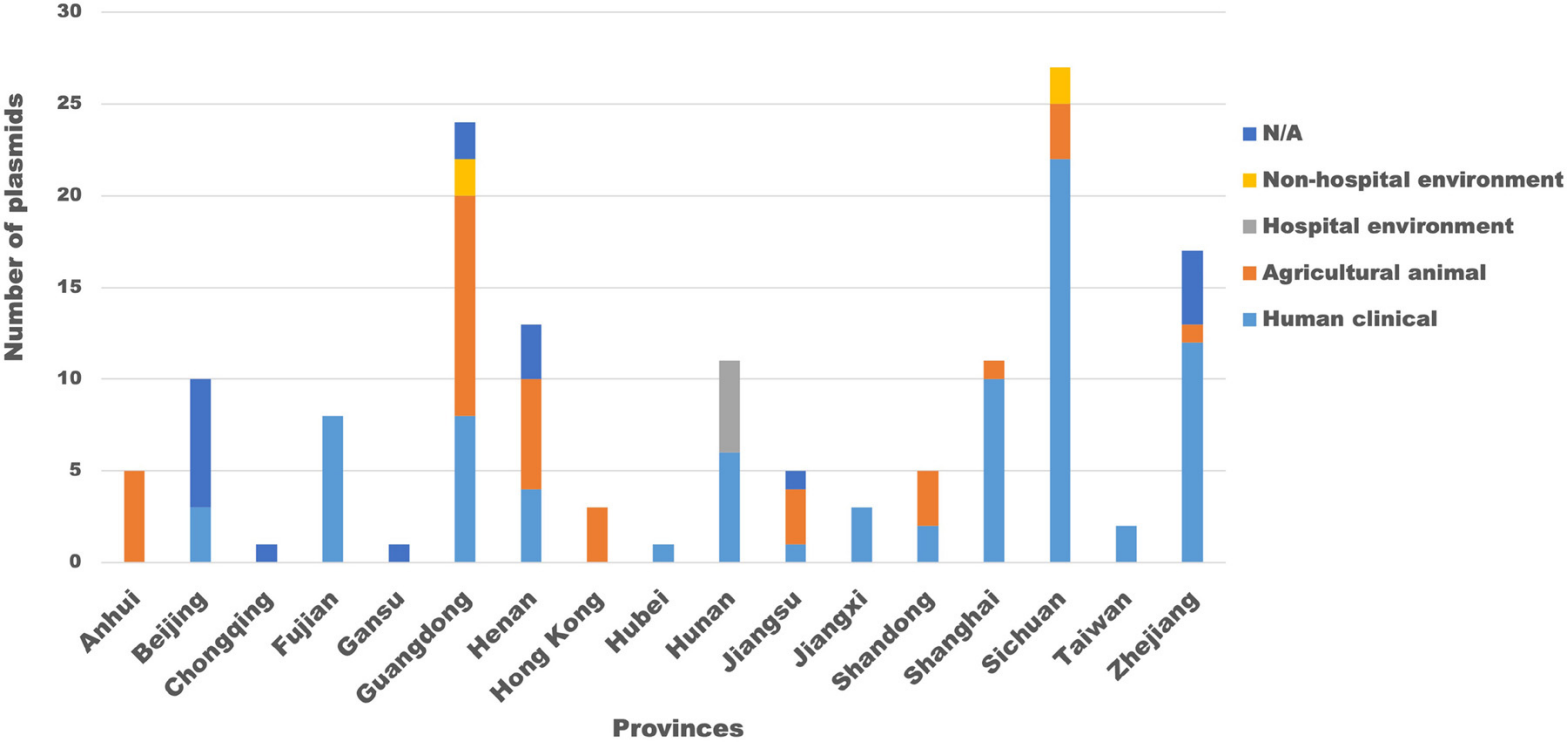
Derivation of bacterial hosts of FII-33 plasmids in China. The derivation of bacterial hosts is quantified in the graph to the left, where the *x* axis lists Chinese provinces and the *y* axis numbers of plasmids. The color legend used for the graph and pie charts is shown to the right of the graph.

The FII-33 replicon was found in 185 complete plasmids in the GenBank nonredundant nucleotide database (see Table S1 in the supplemental material; the 20 plasmids from Sichuan isolates are also included in this set). These ranged in size from 39,248 bp to 284,309 bp and were found in K. pneumoniae (117 plasmids), E. coli (54 plasmids), Salmonella (Enteritidis, 6; Typhimurium, 1; unknown serovar, 2), Citrobacter freundii (2 plasmids), and Escherichia albertii, Escherichia fergusonii, and Proteus mirabilis (1 plasmid each). The hosts of most FII-33 plasmids were isolated in China, with just 9 isolated in Japan, South Korea, Vietnam, Bolivia, Brazil, Canada, or France (Table S1). Within China, FII-33 plasmids were found in 17 different provinces (Fig. 1). Where metadata were available, hosts of FII-33 plasmids were derived from human clinical samples, hospital environments, farm animals, retail meat, healthy pets, or nonhospital environments (Fig. 1, Table S1). This distribution, coupled with the wide range of years of isolation (Table S1), suggests that FII-33 plasmids have been circulating widely in China for at least a decade.

10.1128/mSystems.00831-21.5 TABLE S1 FII-33 plasmids in GenBank. Download Table S1, XLSX file, 0.03 MB. Copyright © 2022 Hu et al. 2022 Hu et al. https://creativecommons.org/licenses/by/4.0/ This content is distributed under the terms of the Creative Commons Attribution 4.0 International license.

### FII-33 plasmids carry a diverse array of antibiotic resistance genes.

**FIG 2:**
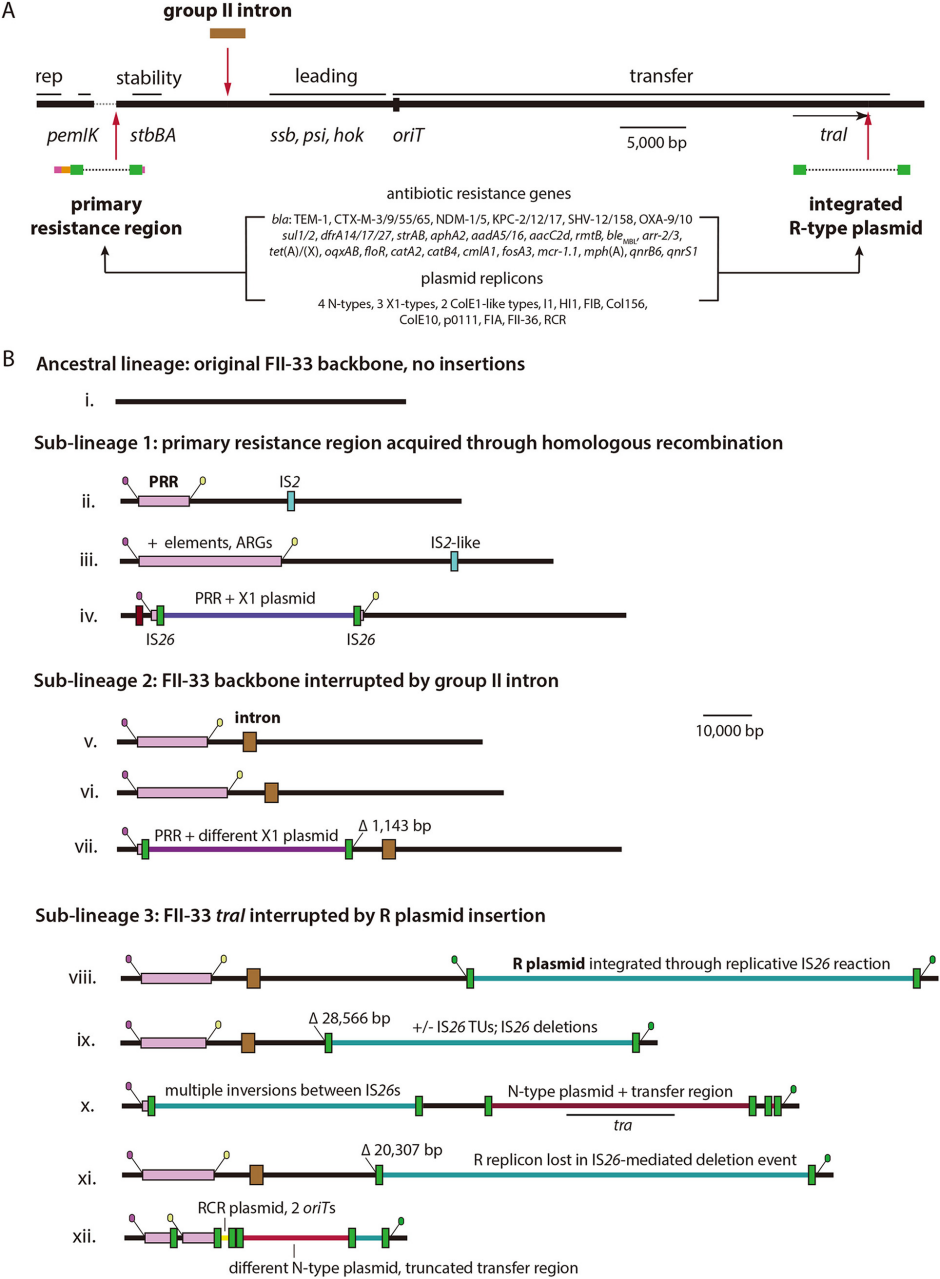
Evolution of FII-33 plasmids. (A) Linear representation of the FII-33 backbone, with replication (rep), stability, leading, and transfer regions labeled above the horizontal line that represents the sequence. The positions of the primary resistance region (PRR), group II intron, and R-type plasmids when present are indicated by labeled vertical arrows. Lists of the antibiotic resistance genes and plasmid replicons that have been acquired within the PRR or R plasmid insertion are listed in the center. The figure was drawn to scale using the sequence of pHN7A8 (GenBank accession no. JN232517) as a reference. (B) Linear maps of FII-33 plasmids chosen to illustrate the evolutionary trajectory of the group. The FII-33 backbone is shown as a black line, while insertions that include sequences from other plasmid backbones are different colors. The PRR, intron, and insertion sequences are shown as labeled colored boxes. Angled lollipops indicate precise translocatable element-backbone junctions flanking the PRR and R plasmid insertion regions. Short descriptions of events responsible for major changes in plasmid configurations are given. The figure is drawn to scale from accession numbers CP055254 (ii), CP017981 (iii), CP050713 (iv), JN232517 (v), KT879914 (vi), CP014139 (vii), CP036372 (viii), CP028796 (ix), CP033394 (x), CP036306 (xi), and CP017086 (xii).

All but one of the FII-33 plasmids examined here contain one or more antibiotic resistance genes, including genes that confer resistance to β-lactams, aminoglycosides, chloramphenicol, fosfomycin, tetracycline, trimethoprim, sulfonamides, rifampicin, quinolones, and colistin (Fig. 2A, Table S2). The carriage of carbapenemase genes is particularly concerning. The globally disseminated *bla*_KPC-2_ gene (31) is found in 103 FII-33 plasmids, while *bla*_KPC-12_ and *bla*_KPC-17_ are found in two and one, respectively (Table S2). KPC-12 exhibits reduced carbapenemase activity relative to KPC-2 (25), but the activity of the KPC-17 enzyme has not been characterized. The metallo-β-lactamase gene *bla*_NDM-1_ is found in two FII-33 plasmids and *bla*_NDM-5_ in one (Table S2). Several extended-spectrum β-lactamase (ESBL) genes, including *bla*_CTX-M-3_, *bla*_CTX-M-14_, *bla*_CTX-M-55_, *bla*_CTX-M-65_, *bla*_SHV-12_, and *bla*_SHV-158_, are also present, while *bla*_TEM-1_ is found in 149 plasmids (Table S2).

10.1128/mSystems.00831-21.6 TABLE S2 Antibiotic resistance genes of FII-33 plasmids. Download Table S2, XLSX file, 0.02 MB. Copyright © 2022 Hu et al. 2022 Hu et al. https://creativecommons.org/licenses/by/4.0/ This content is distributed under the terms of the Creative Commons Attribution 4.0 International license.

Among multiple aminoglycoside resistance determinants, the *rmtB* gene found in 109 FII-33 plasmids (Table S2) is most concerning, as the 16S rRNA methylase it encodes provides high-level resistance to all aminoglycosides used clinically, including amikacin, gentamicin, and tobramycin (32). The *fosA3* gene found in 94 plasmids (Table S2) is also important clinically, as the metallo-glutathione transferase it encodes is responsible for fosfomycin resistance (33). A single FII-33 plasmid contains the *mcr-1.1* gene that confers resistance to the last-resort antibiotic colistin (34). The cooccurrence of resistance determinants in multiple FII-33 plasmids concerningly shows that acquisition of these plasmids can lead to clinically relevant multidrug resistance in a single step.

### All FII-33 plasmids are derived from a single lineage characterized by acquisition of a complex resistance region.

The FII-33 plasmid backbone, first described for pHN7A8 (9), is 61,399 bp and contains a replication region, complete F-like transfer region, a leading region containing genes for establishment in recipients, and a stability region containing genes for partitioning and a toxin-antitoxin system (Fig. 2A). The FII-33 backbone has been interrupted by insertions on several occasions, but three insertions that were each present in greater than 50% of plasmids studied here were used to trace the broad evolution of the group (Fig. 2).

The antibiotic resistance gene region in pHN7A8 containing *bla*_TEM-1_, *bla*_CTX-M-65_, *fosA3*, and *rmtB* is derived from a structure that formed in the context of Tn*2670* in a plasmid closely related to NR1 (5). This primary resistance region (PRR) was acquired by FII-33 plasmids through exchange of the backbone segment containing it by homologous recombination (9). When the Tn*2670*-derived segment was acquired by FII-33 plasmids cannot be determined from the available sequence data. The region must have been acquired in or prior to 2008, when the host of pHN7A8 was isolated from a dog in Guangdong province (9), but the acquisition event may have occurred as early as the 1950s, by which time Tn*2670* was present in NR1 (2). The Tn*2670* region of NR1 has been similarly acquired on at least one other occasion by an FII-18:FIB-1 colicin virulence plasmid (8). This highlights the important role that homologous recombination events can play in the movement of antibiotic resistance determinants between related but compatible plasmid types and the generation of successful, widely disseminated resistance plasmid lineages.

One or both of the 100-bp signature sequences that span the junctions between IS*1* at either end of the Tn*2670*-derived region and the adjacent plasmid backbone were found in all FII-33 plasmids examined here. Each contains a variant of the PRR, indicating that it was acquired before their wide dissemination. It seems likely that the acquisition of this region contributed to the early success of FII-33 plasmids, facilitating their spread.

### Absence of the acquired group II intron is characteristic of a sublineage ancestral to pHN7A8.

The backbone of pHN7A8 is also interrupted by a 2,343-bp group II intron (Fig. 2A), which was inserted in an open reading frame that encodes a 77-amino-acid hypothetical protein. Querying the collection with the intron-backbone junction sequences revealed that 58 plasmids do not contain the group II intron. In these, the intron insertion site is uninterrupted. As the intron-free plasmids also contain the PRR, it seems most likely that the intron was acquired after the PRR (Fig. 2B). If so, intron-free plasmids are ancestral to plasmids like pHN7A8 but have continued to circulate in China after the emergence of the pHN7A8-like sublineage. FII-33 plasmids of the ancestral lineage have been seen in pig- and chicken-associated E. coli and S. enterica in Anhui, Henan, and Sichuan provinces, Hong Kong, and, as recently as 2018, in an E. coli strain isolated from a goose farm in Jiangsu province (GenBank accession no. CP034846) (35). Plasmids of this lineage, which we refer to as sublineage 1, have also been seen in human clinical E. coli isolates in China and in South Korea (Tables S1 and S3).

10.1128/mSystems.00831-21.7 TABLE S3 FII-33 plasmid sublineages, locations, and bacterial hosts. Download Table S3, DOCX file, 0.02 MB. Copyright © 2022 Hu et al. 2022 Hu et al. https://creativecommons.org/licenses/by/4.0/ This content is distributed under the terms of the Creative Commons Attribution 4.0 International license.

### FII-33:R plasmid cointegration occurred once in K. pneumoniae ST11.

The next major insertion in the FII-33 backbone resulted in the formation of a cointegrate comprised of FII-33 and R-type plasmids carrying the *bla*_KPC-2_ carbapenemase gene. This cointegration event was mediated by a replicative IS*26* reaction, generating the 8-bp target site duplication (TSD) CGGGAAAC in the FII-33 *traI* gene (Fig. 2B). Using the IS*26*-backbone junctions at either end of the R plasmid insertion to query the collection revealed that 109 plasmids are FII-33:R cointegrates. However, in 29 of these the R replicon has been lost in IS*26*-mediated deletion events. This highlights the utility of junction sequence queries for subtyping and surveillance of this plasmid group, as replicon typing alone would not have accurately identified these plasmids as part of the FII-33:R sublineage, which we refer to as sublineage 3. In many FII-33:R plasmids, the left or right IS*26*-backbone junction is missing, and closer examination revealed that this is due to the deletion of adjacent FII-33 backbone sequences by IS*26* (Fig. 2B, ix, x, and xi). The precise extents of these deletion events might be used to trace closely related plasmids or link individuals to specific outbreaks. For example, a deletion event that removed 3,031 bp of the interrupted *traI* gene is found in four plasmids from ST11 isolates, pF1_1 and pF127_1 from clinical samples in Fujian province in 2014, an unnamed plasmid (GenBank accession no. CP018455), and pKSH203-KPC from clinical samples in Sichuan province in 2015 and 2018 (Tables S1 and S3). This suggests interprovincial spread of the specific ST11 sublineage in which this deletion event occurred.

All but one sublineage 3 plasmid were found in K. pneumoniae (Table S1 and S3). Where chromosomal sequences were available (*n* = 69), the hosts of these plasmids were typed by multilocus sequence typing, and all were ST11, the most prevalent carbapenem-resistant K. pneumoniae clone in China (16, 36). Given their strong association, it seems likely that the FII-33:R cointegrate formation event occurred in an ST11 isolate. As that cointegration event interrupted the FII-33 relaxase gene and R-type plasmids are nonconjugative (37), abolition of FII-33 transfer ability might explain the apparent confinement of this sublineage to ST11.

### R plasmid cointegration abolished FII-33 plasmid transfer ability.

F-type plasmids with complete, uninterrupted transfer regions are expected to be conjugative. However, the *traI* relaxase gene interrupted by the FII-33:R cointegration event is required for conjugative transfer (38). To determine the effect of the interruption of *traI* on conjugative ability, transfer data were collected for 21 FII-33 plasmids with and without the R plasmid insertion (Table 1).

**TABLE 1:** Transfer properties of FII-33 plasmids

GenBank accession no.	Additional replicon(s)	Notes	Conjugation	
			Expectation	Result
CP026584	R	+Intron, FII-33:R	No	No^*a*^
CP027067	R	+Intron, FII-33:R	No	No^*a*^
CP028541	R	+Intron, FII-33:R	No	No^*a*^
CP028547	R	+Intron, FII-33:R	No	No^*a*^
CP028582	R	+Intron, FII-33:R	No	No^*a*^
CP028796	R	+Intron, FII-33:R	No	No^*a*^
CP029381	R	+Intron, FII-33:R	No	No^*a*^
CP031720	R	+Intron, FII-33:R	No	No^*a*^
CP033404	R	+Intron, FII-33:R	No	No^*a*^
CP036301	R	+Intron, FII-33:R	No	No^*a*^
CP036362	R	+Intron, FII-33:R	No	No^*a*^
CP036372	R	+Intron, FII-33:R	No	No^*a*^
CP028790		+Intron, FII-33:R, *rep* lost	No	No^*a*^
CP036306		+Intron, FII-33:R, *rep* lost	No	No^*a*^
CP036366		+Intron, FII-33:R, *rep* lost	No	No^*a*^
CP038003		+Intron, FII-33:R, *rep* lost	No	No^*a*^
CP033394	N	+Intron, FII-33:R, *rep* lost	Yes^*f*^	Yes^*b*^
CP026576		No intron, no R insert	Yes	Yes^*a*^
MK419152	N, p0111	No intron, no R insert	Yes	Yes^*c*^
KU321583	N, X1	No intron, no R insert	Yes	Yes^*e*^
LN897474		+Intron, no R insert	Yes	Yes^*d*^
LN897475		+Intron, no R insert	Yes	Yes^*d*^

a This study.

b Reported by Qin et al. (25).

c Reported by He et al. (14).

d Reported by Sennati et al. (39).

e Reported under GenBank accession no. KU321583.

f Conjugative ability was restored by the incoming N plasmid, which contains a complete transfer region.

Transfer data for four FII-33 plasmids that lack the R plasmid insertion have been published previously (Table 1). These include two plasmids with and two without the group II intron. pD72c (GenBank accession no. MK419152) is a group II intron-free cointegrate plasmid that has acquired N and p0111-type plasmids within its PRR (14), and pE80 (GenBank accession no. KU321583) is an intron-free FII-33:N:X1 cointegrate with the incoming plasmids in its PRR. The intron-containing plasmids p397Kp (GenBank accession no. LN897474) and p477Kp (GenBank accession no. LN897475) contain no further insertions and belong to the same sublineage as pHN7A8 (39). Thus, FII-33 plasmids with complete and uninterrupted transfer regions, with and without group II introns or additional plasmids inserted in the PRR, are conjugative.

Here, 16 FII-33:R cointegrate plasmids were tested for conjugative ability by mating their K. pneumoniae ST11 hosts with E. coli J53. Four of these cointegrates had lost the R plasmid replicon in IS*26*-mediated deletion events within the R plasmid insert region but retained the signatures of R plasmid insertion (Table 1). None of these plasmids were transferrable, indicating that the interruption of *traI* by the R plasmid insertion event has abolished FII-33 plasmid transfer ability. This supports the hypothesis that loss of transfer ability has resulted in the host restriction of the FII-33:R sublineage.

The sublineage 3 plasmid pCTXM65_015625 (GenBank accession no. CP033394) has lost the R replicon but includes an additional N-type replicon. Despite having an interrupted *traI* gene, pCTXM65_0156625 has recently been shown to transfer from K. pneumoniae ST11 to E. coli J53 at low frequencies (25). Examination of pCTXM65_0156625 revealed that it is a triple cointegrate, having acquired an N-type plasmid through a conservative IS*26*-mediated event. The N-type plasmid, which was inserted within the R plasmid region of pCTXM65_0156625, includes a complete transfer region (Fig. 2B, x), and it appears that this region has restored transfer ability.

Only a single plasmid from the FII-33:R sublineage has been seen outside K. pneumoniae (Table S3). pT18 from a clinical P. mirabilis isolate has lost the R-type replicon but acquired an N-type plasmid and a small pBuzz-like rolling circle plasmid in further cointegration events (13). The N-type transfer region in pT18 has been truncated in an IS*26*-mediated deletion event, and pT18 is not expected to be conjugative. However, the pBuzz-like region contains two putative *oriT* sites and might render pT18 mobilizable by L- and M-type conjugative plasmids through a relaxase-in *trans* mechanism (13). Therefore, the escape of pT18 from K. pneumoniae ST11 might be explained by conjugation mediated by an N-type transfer region that was subsequently lost or by mobilization mediated by the pBuzz-like plasmid *oriT* mimics and a coresident conjugative plasmid.

### Loss of FII-33 backbone sequences in cointegrate plasmids.

Of 109 FII-33:R cointegrate plasmids, 29 retain the left and right TSD sequences found on either side of the inserted R plasmid. Querying all plasmids with the pHN7A8 *traI* gene revealed that the remaining FII-33:R cointegrates had lost one or both of the TSD sequences along with all or part of the interrupted *traI* in deletion events that were mediated by IS*26*. Deletions to the left of the insertion were seen in 77 plasmids, while deletions to the right were seen in three (Table S1). One plasmid had lost both TSDs (GenBank accession no. MN842292). The extents of deletions to the left of the insertion varied from a 448-bp deletion within *traI* (GenBank accession no. MF168404) to deletions of greater than 20 kb that removed the majority of the transfer region (Fig. 2B, ix, x, and xi) or of up to 51.1 kb that removed the entire transfer region as well as the leading region and group II intron (Fig. 2B, xii).

Given the insertion in *traI* appears to abolish transfer ability in FII-33:R cointegrates, the region encoding transfer-associated proteins presumably serves no purpose. As a transfer region with an interrupted relaxase gene would still be expected to produce conjugative pili, expression of these determinants might even be deleterious for the cointegrate plasmid’s host. Conjugative pili serve as receptors for some types of bacteriophages (40), and presentation of these on the host cell surface might be particularly deleterious when they are not ameliorating this disadvantage by enabling horizontal gene transfer. Deletions that remove nonfunctional transfer region segments would therefore be advantageous and might be selected for.

### Cointegration is common and might modify plasmid traits.

To detect further cointegrates, all 185 FII-33 plasmids were tested for the presence of additional replicons using the PlasmidFinder database. Additional replicons were found in 47 plasmids and included four different N-types, three different X1-types, three different ColE1-like types, and FII, FIA, FIB, I1, rolling-circle, and Col156 types (Table S3). That FII-33 plasmids have formed cointegrates with such a variety of different plasmids and, in the case of N and X1-type plasmids, with multiple variants of the same types suggests that cointegration events are more common than is currently appreciated.

Plasmids containing additional replicons were examined manually, and in all cases the acquired replicons were found within the PRR or R-plasmid insert. Sequences acquired in cointegration events were always flanked by IS*26*, which seems likely to have been responsible for cointegration. No target site duplications were found flanking acquired sequences, which is consistent with cointegrate formation through the targeted conservative mechanism that requires the presence of IS*26* copies in both participating molecules and does not generate an additional IS*26* or a target site duplication (41).

In a few cases, the acquisition of particular replicons can be tied to the acquisition of antibiotic resistance genes. For example, the *aadA17*-*dfrA5* gene cassettes and *aacC2d* are only found in p283149-FII (GenBank accession no. MN823989) and appear to have been acquired along with the FIB and Col156 replicons that are not shared by any other FII-33 plasmid studied here. pD72c, derived from a pig fecal sample collected in Henan province and described above, acquired the colistin resistance gene *mcr-1.1* along with a p0111-type P1-like phage plasmid in a single IS*26*-mediated cointegration event. This event involved two naturally occurring plasmids and occurred in the laboratory, while the original host was involved in a conjugation experiment (14). Together, these examples show that cointegration contributes directly to the accumulation of antibiotic resistance determinants and can occur over short periods of time.

Cointegrate formation might influence plasmid behavior. Some incoming plasmids have contributed complete transfer regions or *oriT* sequences as described above, possibly restoring transfer ability after FII-33 backbone interruptions or deletions. Other acquired plasmids have introduced additional toxin-antitoxin or partitioning genes that contribute to plasmid stability. Additional replicons might allow the cointegrates to replicate in species that FII-33 plasmids cannot normally replicate in, potentially increasing their host range. Experimental tests will be required to determine the host ranges and stability of FII-33 plasmids alone and in cointegrate molecules.

### Resources for rapid identification and subtyping of FII-33 plasmids.

Resources are provided here for identifying and subtyping FII-33 plasmids in complete or draft genome sequences. Text S1 contains the FII-33 *repA1* gene, the FII-33 sequence from PubMLST, and signature sequences that allow detection of the PRR, group II intron, and R plasmid insertions, facilitating subtyping of plasmids into each of the major sublineages. Further characteristics specific to sublineages of interest or newly emerged sublineages can be targeted with the same approach. The backbone sequence of pHN7A8 is also included in Text S2, and the contents of the backbone are described in Text S3. Comparison to this backbone sequence will allow the extent and content of backbone deletion events to be determined. Replicon typing using the PlasmidFinder database would allow identification of integrated plasmid replicons. A guide to typing FII-33 plasmids to the sublineage level is included in Text S3, and a simplified overview of the events that generated sublineages 1, 2, and 3 is shown in Fig. S1. We have recommended using stringent sequence identities to avoid misidentification of distinct lineages such as FII-40, FII-55, FII-63, or FII-73 that differ from FII-33 by two to five single-nucleotide polymorphisms (SNPs) in the 156-bp sequences targeted by PubMLST but outline some caveats to this in Text S3.

10.1128/mSystems.00831-21.1 TEXT S1 The FII-33 *repA1* gene, the FII-33 sequence from PubMLST, and signature sequences. Download Text S1, RTF file, 0.001 MB. Copyright © 2022 Hu et al. 2022 Hu et al. https://creativecommons.org/licenses/by/4.0/ This content is distributed under the terms of the Creative Commons Attribution 4.0 International license.

10.1128/mSystems.00831-21.2 TEXT S2 The backbone sequence. Download Text S2, RTF file, 0.06 MB. Copyright © 2022 Hu et al. 2022 Hu et al. https://creativecommons.org/licenses/by/4.0/ This content is distributed under the terms of the Creative Commons Attribution 4.0 International license.

10.1128/mSystems.00831-21.3 TEXT S3 Typing FII-33 plasmids. Download Text S3, DOCX file, 0.02 MB. Copyright © 2022 Hu et al. 2022 Hu et al. https://creativecommons.org/licenses/by/4.0/ This content is distributed under the terms of the Creative Commons Attribution 4.0 International license.

10.1128/mSystems.00831-21.4 FIG S1 A simplified overview of the events that generated sublineages. Download FIG S1, PDF file, 0.4 MB. Copyright © 2022 Hu et al. 2022 Hu et al. https://creativecommons.org/licenses/by/4.0/ This content is distributed under the terms of the Creative Commons Attribution 4.0 International license.

**FIG 3:**
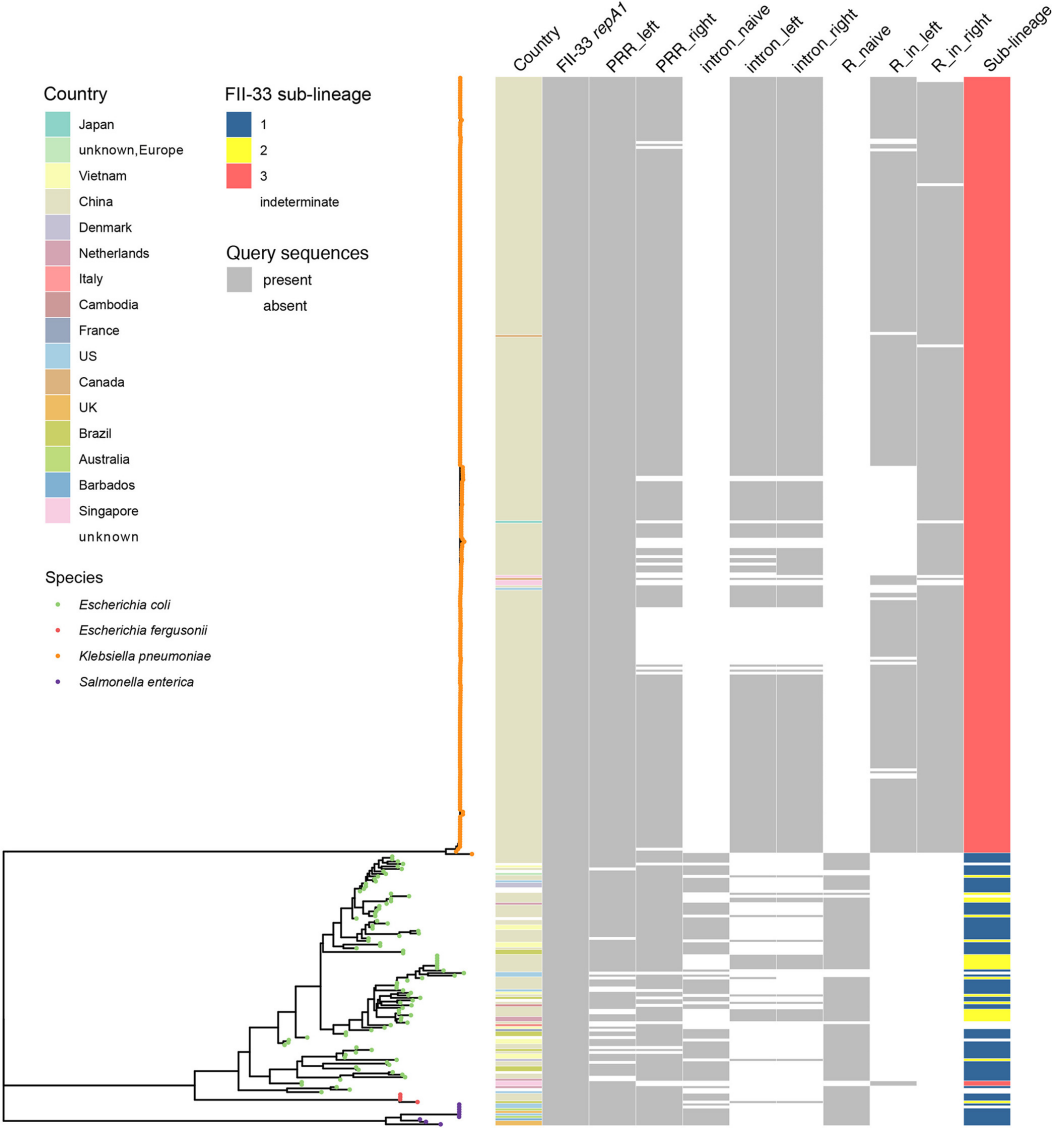
Detection and subtyping of plasmids in draft genome sequences. Neighbor-joining tree drawn from 423 core genomes that contain the FII-33 *repA1* gene. See Materials and Methods for details. Alongside the tree is source of isolation data and presence/absence data for each of the FII-33 subtyping signature sequences as well as sublineage classifications for the plasmids in each genome. The species of each genome is indicated by a colored dot in the tree, and presence/absence and subtyping data are shown as colored blocks, with a color legend shown to the left of the tree.

To demonstrate the utility of our approach, we searched for and subtyped FII-33 plasmids in a curated collection of 661,405 bacterial draft genome sequences (27). The FII-33 *repA1* gene was detected in 423 genomes. The presence or absence of signature sequences associated with the PRR, group II intron, or R plasmid insertion were used to determine that plasmids of sublineage 1, sublineage 2, and sublineage 3 were present in 77, 22, and 314 genomes, respectively (Fig. 3). Ten genomes lacked the naive intron insertion site as well as the left and right intron-backbone junctions, so whether they contained plasmids of sublineage 1 or sublineage 2 could not be determined. The majority of FII-33 plasmids were found in genomes from China, although their presence in 55 genomes from 15 further countries is indicative of wider international dissemination than was revealed by complete plasmid sequences in GenBank. As expected, all but two sublineage 3 plasmids were found in closely related K. pneumoniae ST11, while sublineage 1 and 2 plasmids were found in a diverse set of E. coli, *E. fergusonii*, and S. enterica.

### Surveillance of FII-33 plasmids.

FII-33 plasmids clearly play an important role in the dissemination of antibiotic resistance genes in China. It will be important to understand where these plasmids are transferring, forming cointegrates, and accumulating resistance determinants if preventative action is to be taken. The analysis presented here has been facilitated by sequencing efforts in China that have focused on bacterial isolates collected from clinical specimens, healthy humans and animals, hospitals, farms, and the environment. All of these sources have yielded FII-33 plasmids, emphasizing the importance of monitoring often-overlooked reservoirs of antibiotic resistance determinants.

It is clear that plasmids must be considered when integrating whole-genome sequence data with infection control and prevention strategies that aim to prevent the spread of antibiotic resistance determinants within and between hospitals (42). Surveillance networks sensitive enough to detect problematic plasmid sublineages will be essential if such strategies are to be effective. However, the output of commonly used basic typing tools such as PlasmidFinder or MOBsuite do not provide adequate resolution to determine whether plasmids called the same type are in fact members of the same lineage. Using specific signature sequences like the ones described here and recently for plasmids of the L/M complex (43) might enable rapid detection of important locally or globally disseminated sublineages. Although the generation of informative signature sequences requires detailed analysis, once created they can be shared and applied to draft or complete genome sequences with relatively simple BLAST commands and output interpretation. An extensive database of frequently updated signatures combined with more comprehensive basic typing databases might be designed such that it can be applied without the need for specialized knowledge of plasmid evolution.

It is hoped that the tools provided here will allow other groups to rapidly subtype FII-33 plasmids and report on the spread of specific sublineages. It will be particularly interesting to trace any further appearances of FII-33 plasmids outside China. Although it is known that plasmid lineages disseminate internationally, the routes of their dispersal are very difficult to track due to incomplete sampling and an inability to rapidly type plasmids to the sublineage level. Although the sampling problem cannot be easily solved, our understanding of FII-33 plasmids can now facilitate tracing them at global scales where sequence data are available.

### Conclusions.

FII-33 plasmids are widely distributed in agricultural, commensal, and clinical isolates in China and have likely been circulating in human-associated bacterial populations for decades. They carry an antibiotic resistance region derived from one that formed in a different F-type plasmid in or prior to the 1950s, and acquisition of this region likely contributed to their successful dissemination. In the time since the PRR was acquired, FII-33 plasmids have formed multiple sublineages distinguished by insertion or cointegration events. All sublineages have continued to accumulate antibiotic resistance genes. Most concerningly, a cointegrate FII-33:R sublineage that emerged in K. pneumoniae ST11 carries the *bla*_KPC-2_ carbapenemase gene and is already widely distributed and seemingly entrenched in hospital-associated populations.

Broadly, this study provides important insight into the ongoing evolution of antibiotic resistance plasmids. Where previously we have seen the emergence and dissemination of relatively simple plasmids consisting of individual backbones interrupted by simple or complex insertions, cointegrate plasmids are becoming increasingly common in clinical isolates. If cointegrate plasmids are to become prominent in antibiotic-resistant bacterial pathogens, it will be important for us to understand their properties and evolutionary trajectories.

## MATERIALS AND METHODS

### Strain collection, genome sequencing, and analysis.

Seventeen K. pneumoniae strains and three E. coli strains were all recovered from clinical samples that were collected in Sichuan province between 2014 and 2018. All 20 isolates were subjected to whole-genome sequencing using HiSeq X10 (Illumina; San Diego, CA, USA) according to the manufacturer’s instructions. Genomic DNA was prepared using the QIAamp DNA minikit (Qiagen, Hilden, Germany). Generated reads were *de novo* assembled into contigs using SPAdes (17), applying the careful and auto-cutoff modes. Five of them were also subjected to long-read whole-genome sequencing using a MinION Sequencer (Nanopore; Oxford, UK). The *de novo* hybrid assembly of both short (Illumina) and long reads was performed using Unicycler (18) under conservative mode for increased accuracy. Pilon (19) was used to correct complete circular contigs with Illumina reads for several rounds until no change was detected.

### Plasmid sequences examined.

The 156-bp sequence that represents the FII-33 replicon in the PubMLST plasmid typing database (https://pubmlst.org/organisms/plasmid-mlst) was used to query the GenBank nonredundant nucleotide database (last search, 30 June 2020), and complete plasmids with matches identical to the sequence were included in this study. All plasmids were opened in the same orientation at the same position 648 bp upstream of the *repA1* replication initiation gene.

### Plasmid sequence analysis.

Plasmid replicons were identified using PlasmidFinder (20) (https://cge.cbs.dtu.dk/services/PlasmidFinder/), antibiotic resistance genes using ResFinder (21) (http://cge.cbs.dtu.dk/services/ResFinder/), and insertion sequences using ISFinder (22) (https://isfinder.biotoul.fr/). Gene Construction kit v4.5 (Textco Biosoftware, Raleigh, NC) was used to visualize and manually annotate plasmid sequences. Plasmid sequences listed in Table S1 in the supplemental material were compiled into a nucleotide database and queried using standalone BLAST (23) with informative sequences selected from annotated plasmids.

### Conjugation experiments.

Conjugation experiments were carried out in blood heart infusion broth (Oxoid, Hampshire, United Kingdom) and on nitrocellulose filters (GE Life Science, Pittsburgh, PA, United States) at both 30°C and 37°C, as described previously (24). Sodium azide-resistant E. coli strain J53 was used as the recipient. For the broth method, the donor and recipient were mixed at a ratio of 1:10, and the mixture was incubated overnight. For the filter method, the donor and recipient were mixed at a ratio of 1:1, and the mixture was incubated for 4 h. Transconjugants were selected on LB agar plates containing 2 μg/ml meropenem and 150 μg/ml sodium azide or on LB agar plates containing 16/4 μg/ml piperacillin-tazobactam and 150 μg/ml sodium azide. Strain 015625, containing conjugative plasmid pCTXM65_015625 (25), served as a positive control, and suspected transconjugants were confirmed by PCR with primers F33L/R, 5′-CCGAAAAGGTAATCCTCTGA/ACAAACAGGCAAGAATGTGA, CTXMF/R, 5′-AGTGCAACGGATGATGTTCG/TTCTGCCAGCGTCATTGTG, and J53-unique1F/R, 5′-ACGGACTAACAGCCTGGAAA/TAGCGTATCCAGCGTCACTT.

### Identifying and typing FII-33 plasmids in a draft genome collection.

The sequences in Text S1 were used to query the COBS index (26) of 661,405 curated draft genomes (27) with a kmer similarity cutoff of 1.00 such that only identical sequences were detected. The estimated core genome distances of the 423 genomes that contained the FII-33 *repA1* gene were extracted from the pp-sketch index of the 661,405-genome collection (27) using pp-sketchlib (version 1.5.1) (28). A neighbor-joining tree of the core distances was produced using RapidNJ (version 2.3.2) (29), and the tree, metadata, and query sequence presence/absence data were visualized in R using the ggtree package (30). The figure was edited manually, and sublineage identifications were added using Adobe Illustrator.

### Data availability.

The 20 complete FII-33 plasmids from strains isolated in Sichuan province have been deposited in GenBank under accession numbers CP025949, CP026576, CP026584, CP027067, CP028541, CP028547, CP028582, CP028790, CP028796, CP029381, CP031720, CP033394, CP033404, CP036180, CP036301, CP036306, CP036362, CP036366, CP036372, and CP038003.
