# Supplementary material for: Fine-Scale Reconstruction of the Evolution of FII-33 Multidrug Resistance Plasmids Enables High-Resolution Genomic Surveillance
Source: mSystems. 2022 Jan 18;7(1):e00831-21. doi: 10.1128/msystems.00831-21 (PMC8765060; doi:10.1128/msystems.00831-21)
Supplement: TABLE S3 [file msystems.00831-21-st003.docx]

**Table S3:** FII-33 plasmid sub-lineages, locations and bacterial hosts

| Lineage | Definition | Sub-lineage | Replicon  gain/loss | #  plasmids | Locations | Hosts |
| --- | --- | --- | --- | --- | --- | --- |
| **1** | + PRR | 1.1 | - | 19 | Anhui, Guangdong, Henan, Hong Kong, Jiangsu, Sichuan, Brazil, South Korea | *E. coli*  *S. enterica*  *K. pneumoniae* |
|  |  | 1.2 | + N/X1/ColE10 | 35 | Anhui, Beijing, Guangdong, Henan, Hong Kong, Shandong, Shanghai, Sichuan, Zhejiang, Vietnam, Japan | *E. coli*  *E. albertii*  *S. enterica*  *K. pneumoniae*  *C. freundii* |
|  |  | 1.3 | + N/X1/ColE10  + p0111 or HI1 | 2 | Henan | *E. coli* |
|  |  | 1.4 | + FII-36 + FIA | 1 | China | *E. coli* |
|  |  | 1.5 | + FIB + Col156 | 1 | Guangdong | *K. pneumoniae* |
| **2** | + PRR  + intron | 2.1 | - | 15 | Anhui, Guangdong, Henan, Shandong, Zhejiang, Bolivia | *E. coli*  *E. fergusonii*  *E. cloacae*  *K. pneumoniae*  *C. freundii* |
|  |  | 2.2.1 | + X1 | 1 | Jiangsu | *E. coli* |
|  |  | 2.2.2 | + X1 + X1 | 1 | Sichuan | *K. pneumoniae* |
| **3** | + PRR  + intron  + R in *traI* | 3.1 | - | 74 | Beijing, Chongqing, Fujian, Guangdong, Henan, Hubei, Hunan, Jiangsu, Jiangxi, Shandong, Sichuan, Canada | *K. pneumoniae* |
|  |  | 3.1.2 | + N-type ‘a’ | 1 | Taiwan | *K. pneumoniae* |
|  |  | 3.1.3 | + N-type ‘b’ | 1 | Henan | *K. pneumoniae* |
|  |  | 3.1.4 | + ColRNAI-type ‘a’ | 1 | China | *K. pneumoniae* |
|  |  | 3.1.5 | + ColRNAI-type ‘b’ | 1 | Beijing | *K. pneumoniae* |
|  |  | 3.1.6 | + I1 | 1 | Zhejiang | *K. pneumoniae* |
|  |  | 3.2.1 | R replicon lost | 27 | Beijing, Fujian, Gansu, Guangdong, Jiangxi, Sichuan, Taiwan, Zhejiang, Canada | *K. pneumoniae* |
|  |  | 3.2.2 | + N-type ‘a’  R replicon lost | 1 | Sichuan | *K. pneumoniae* |
|  |  | 3.2.3 | + N-type ‘b’  R replicon lost | 1 | China | *K. pneumoniae* |
|  |  | 3.2.4 | R replicon lost  + N-type ‘c’  + pBuzz-like RCR | 1 | Zhejiang | *P. mirabilis* |

Plasmids that were not typed:

MT230416 – contains partial translocatable element sequences that cannot be explained by transposition or deletion events, and are therefore indicative of sequence assembly issues. This plasmid was excluded from the study.

MK416152 – Contains the 156 bp sequence upstream of *repA1* that is used to identify FII-33 plasmids by PubMLST, but does not contain the FII-33 *repA1*. This is likely a recombinant plasmid that has acquired the FII-33 replication regulation region but has an otherwise different backbone. It was therefore excluded.
