# Supplementary material for: Fine-Scale Reconstruction of the Evolution of FII-33 Multidrug Resistance Plasmids Enables High-Resolution Genomic Surveillance
Source: mSystems. 2022 Jan 18;7(1):e00831-21. doi: 10.1128/msystems.00831-21 (PMC8765060; doi:10.1128/msystems.00831-21)
Supplement: TEXT S3 [file msystems.00831-21-t0003.docx]

**Typing FII-33 plasmids**

The following is a guide to identifying and sub-typing FII-33 plasmids present in complete or draft genome sequences. Genomes should be queried with the provided sequences using BLAST. Results should be interpreted as below.

Steps 1 and 2 of this guide should be sufficient for identifying FII-33 plasmids and typing them to sub-lineage level, while further analysis described in steps 3 and 4 allow for the characterisation of backbone deletions, inversions or substitutions. As it is impossible to predict which translocatable elements might be acquired by FII-33 plasmids, or which configurations they might be found in, manual annotation of insertion regions on a case-by-case basis is advised.

**Step 1: detection of the FII-33 replicon**

The 156 bp sequence used to represent the FII-33 replicon in PubMLST and the 870 bp sequence of the FII-33 replication initiation gene *repA1* are included here. These can be used to identify FII-33 plasmids. It is expected that FII-33 plasmids will contain sequences identical to these, though caution is advised and we recommend using both sequences and considering the presence of FII-33 sub-lineage signatures where results are unclear.

Sequences in the PubMLST database that differ from the 156 bp FII-33 allele by just two (FII-55), three (FII-40), four (FII-63) or five (FII-73) SNPs can be found in plasmids with *repA1* genes that are just 90-94% identical to the FII-33 *repA1*. These plasmids do not contain any of the sequence junctions that define FII-33 sub-lineages and clearly represent distinct lineages that should not be confused with FII-33. For this reason, we strongly recommend using a stringent 100% identity threshold for identifying FII-33 replicons with the 156 PubMLST sequence, or only considering plasmids with divergent 156 bp sequences part of the FII-33 lineage when their *repA1* is identical to the reference and they contain multiple sub-typing sequence junctions.

It is possible that FII-33-derived plasmids will acquire divergent *repA1* alleles through recombination. In these cases, the 156 bp PubMLST sequence and/or the presence of sub-typing junctions should be relied on for identification.

Finally, we cannot rule out the existence of FII-33 cointegrate plasmids that have lost the FII-33 replicon in deletion events. In these cases, only the intron or R-plasmid insertion junctions can provide evidence for the presence of FII-33-derived sequences in the cointegrate. Ultimately, we recommend careful consideration of complete plasmid sequences in all cases where the results of BLAST queries using sequences provided here are unclear.

**Step 2: FII-33 sub-lineage typing**

Once the FII-33 replicon has been detected in a genome, that genome should be queried with the signature sequences that target the primary resistance region (PRR), the group II intron and the R plasmid insertion. The presence or absence of these insertions can be used to assign FII-33 plasmids as below:

| **FII-33 sub-lineage** | **PRR** | **group II intron** | **R plasmid insertion** |
| --- | --- | --- | --- |
| **1** | present | absent | absent |
| **2** | present | present | absent |
| **3** | present | present | present |

Note that all sequences described below are 100 bp long and target precise sequence junctions indicative of the presence or absence of insertions. Only complete, contiguous matches to these queries should be considered positive results.

Detection of the PRR:

Two sequences, PRR-left and PRR-right, are used for detection of the PRR. The sequences span the IS*1*-backbone junctions at the left and right ends of the PRR, including 50 bp of IS*1* and 50 bp of adjacent backbone sequence each. Complete matches to one or both of these sequences indicates that the PRR is present. Note that deletion events can remove these junction sequences, so it is possible that one or both will be absent from a given plasmid. It should also be noted that the PRR-right junction represents the original insertion position of Tn*2670* in an FII-type backbone and will also be found in plasmids like NR1 or others, like pCERC3, that have acquired derivatives of the Tn*2670* region via homologous recombination.

Detection of the group II intron:

Two sequences, intron-left and intron-right, are used for detection of the group II intron insertion. The sequences span the intron-backbone junctions at the left and right ends of the intron, including 50 bp of intron and 50 bp of adjacent backbone sequence each. Complete matches to one or both of these sequences indicates that the PRR is present.

A third sequence, intron-naive, is used for detection of backbones uninterrupted by the group II intron. This sequence spans the intron insertion site as it appears when the intron is not present. A complete match to this sequence indicates that the intron is not present. It is expected that where the intron-naive sequence is present, the intron-left and intron-right sequences will not be present, and where the intron-left and/or intron-right sequences are present, the intron-naive sequence will be absent.

| **intron-naive** | **intron-left** | **intron-right** | **interpretation** |
| --- | --- | --- | --- |
| + | - | - | intron absent |
| - | + | + | intron present |
| - | + | - | intron present |
| - | - | + | intron present |

Detection of the R plasmid insertion:

Two sequences, R-in-left and R-in-right, are used for detection of the R plasmid insertion in *traI*. The sequences span the IS*26*-backbone junctions at the left and right ends of the insertion, including 50 bp of intron and 50 bp of adjacent backbone sequence each. Each of the sequences includes a copy of the target site duplication sequence CGGGAAAC. Complete matches to one or both of these sequences indicates that the R plasmid insertion has occurred. Note that IS26-mediated deletions to the left or right of the R plasmid insertion region are common, so one or both of these signature sequences might be absent from sub-lineage 3 plasmids.

A third sequence, R-naive, is used for detection of backbones uninterrupted by the R plasmid insertion. This sequence spans the intron insertion site as it appears when the *traI* gene has not been interrupted by the insertion of the R-type plasmid. A complete match to this sequence indicates that the R plasmid insertion is not present. It is expected that where the R-naive sequence is present, the intron-left and intron-right sequences will not be present, and where the intron-left and/or intron-right sequences are present, the R-naive sequence will be absent. In cases where all three sequences are absent, the plasmid should be checked for the presence of an R-type replicon using PlasmidFinder. If the replicon is present, the likely explanation is that both the left and right junction sequences have been lost in deletion events.

**Step 3: FII-33 backbone**

Use the FII-33 backbone sequence provided to query the draft or complete genome of interest to determine:

- which parts of the backbone are present/absent
- the potential significance of any absences (refer to the table below)
- whether the backbone has been interrupted by additional insertions

In complete plasmid sequences, it will also be possible to examine the orientation of backbone segments relative to the reference in order to determine whether parts of the backbone have been inverted in IS-mediated events.

Table S4: Overview of FII-33 backbone content

| Position  in reference | Feature^1^ | Notes |
| --- | --- | --- |
| 649-1518 | *repA1* | FII-33 replication initiation gene |
| 3203-3794 | *pemIK* | toxin-antitoxin genes, contribute to plasmid stability |
| 3987-3988 | PRR | primary resistance region located between these bases |
| 6187-7502 | *stbBA* | partitioning genes, contribute to plasmid stability |
| 11932-11933 | intron | group II intron inserted between these bases sub-lineage 2/3 |
| 15839-16366 | *ssb* | single-stranded DNA binding protein determinant |
| 18810-19960 | *psiBA* | SOS inhibition genes |
| 20182-20398 | *hok*/*sok* | toxin-antitoxin genes |
| 23437-23760 | *oriT* | origin-of-transfer; beginning of transfer region |
| 23762-56296 | *tra/trb* | contains all transfer genes from *traM* to *traI* |
| 56142-56143 | R plasmid | R plasmid inserted between these bases in sub-lineage 3 |
| 57117-57677 | *finO* | fertility inhibition gene; end of transfer region |

^1^ To determine precisely which genes are present/absent, compare to the annotated sequence at GenBank accession JN232517. Note that gene co-ordinates in the backbone sequence provided here do not correspond to those in the GenBank entry, which includes a complete plasmid sequence. Consult PMID 7915817 for further information on the function of genes in the transfer region.

**Step 4: characterisation of additional backbone insertions**

If further backbone insertions are detected, determine whether they are flanked by target site duplications. Signature sequences can be created by taking 50 bp of the inserted element and 50 bp of adjacent backbone sequence from either end of the insertion. These can be used to detect more closely-related plasmids in sequence databases that share the insertion of interest.

**Step 5: detection of additional plasmid replicons**

For complete plasmids, the PlasmidFinder database can be used to detect additional plasmid replicons that would indicate that the plasmid of interest is a cointegrate. Note that the detection of additional replicons in a draft genome does not confirm the existence of a cointegrate, so cointegrate formation can only be assessed using complete genome or plasmid sequences.
