# Supplementary material for: Fine-Scale Reconstruction of the Evolution of FII-33 Multidrug Resistance Plasmids Enables High-Resolution Genomic Surveillance
Source: mSystems. 2022 Jan 18;7(1):e00831-21. doi: 10.1128/msystems.00831-21 (PMC8765060; doi:10.1128/msystems.00831-21)
Supplement: FIG S1 [file msystems.00831-21-sf001.pdf]

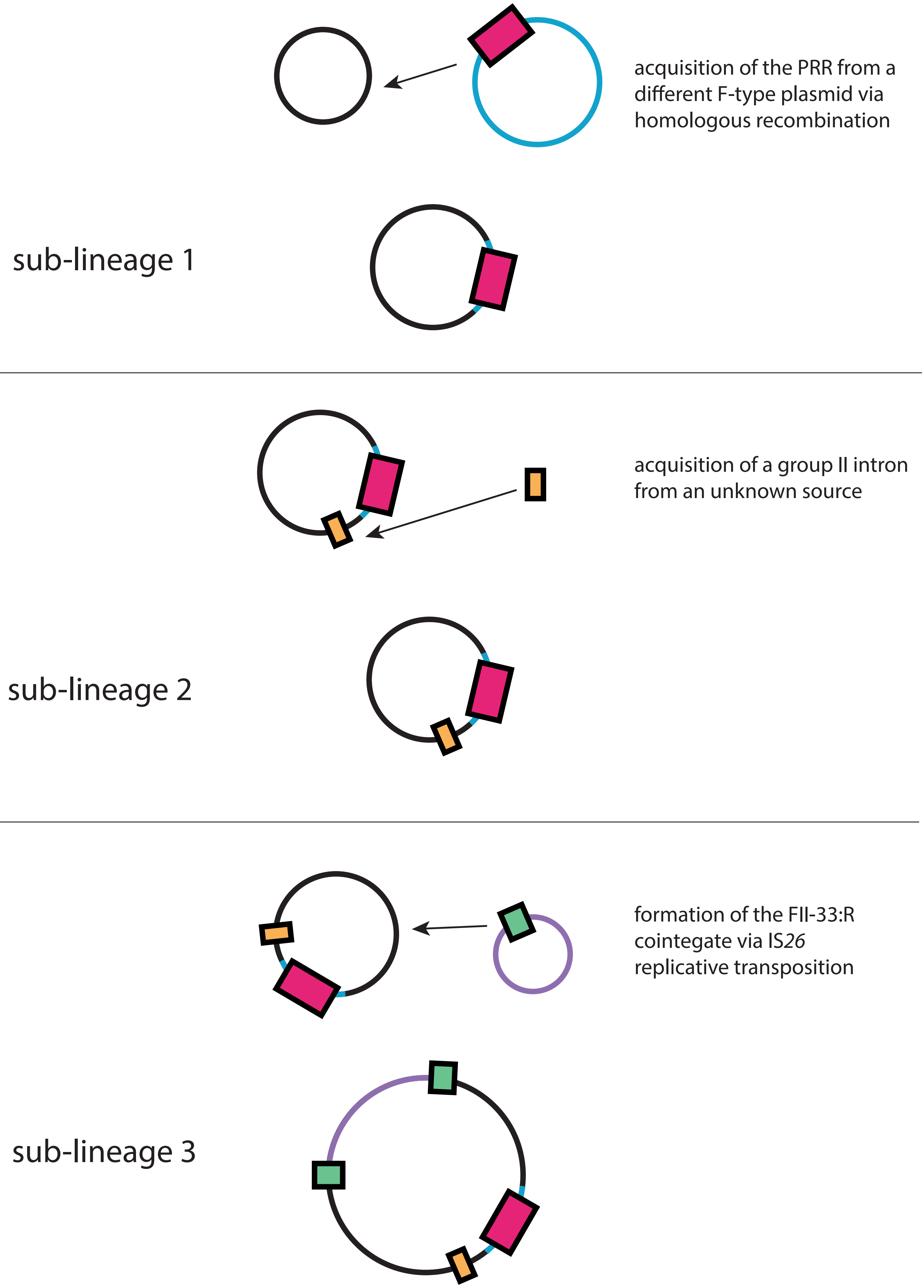
acquisition of the PRR from a different F-type plasmid via homologous recombination
sub-lineage 1
acquisition of a group II intron from an unknown source
sub-lineage 2
formation of the FII-33:R cointegate via IS*26* replicative transposition
sub-lineage 3
